# Development of Reinforced Polyester/Graphene Nanocomposite Showing Tailored Electrical Conductivity

**DOI:** 10.3390/polym12102358

**Published:** 2020-10-14

**Authors:** Federico Serenari, Milad Madinehei, Nima Moghimian, Davide Fabiani, Eric David

**Affiliations:** 1 Department of Electrical, Electronics and Information Engineering (DEI) ‘G. Marconi’, Università Di Bologna, Viale del Risorgimento 2, 40136 Bologna, Italy; federico.serenari@studio.unibo.it (F.S.); davide.fabiani@unibo.it (D.F.); 2 Mechanical Engineering Department, École de technologie supérieure, 1100 Notre-Dame St W, Montréal, QC H3C 1K3, Canada; miladmadinehei@gmail.com; 3 NanoXplore Inc., 4500 Boulevard Thimens, Saint-Laurent, QC H4R 2P2, Canada; Nima.Moghimian@nanoxplore.ca

**Keywords:** polyester composites, graphene, glass fiber, electrical properties, dynamic mechanical analysis

## Abstract

Production process was chosen in order to be readily scalable at the industrial level. The resin/graphene mixture was prepared through high shear mixing at six different weight concentrations between 0% and 10%. Samples were subsequently produced by compression molding. The electrical properties were measured both in-the-plane and across-the-plane using, respectively, a four-point probe and a two-electrode system. The two-electrode system was a dielectric spectrometer, and accordingly, the across-the-plane measurements were conducted in the frequency-domain. Mechanical measurements were conducted using conventional three-point bending and impact setups. The percolation threshold was found to be in the range of 3–5 wt.% concentration, for which the conductivity showed a 7 orders of magnitude increase. These results were quite similar to the samples containing around 50 wt.% of glass fibers. Surprisingly, the in-the-plane conductivity was found to be lower than the bulk conductivity, contrary to what was found with the same filler for thermoplastic composites prepared by melt compounding. No significant increase in mechanical properties as a function of filler loading was observed, except maybe a slight increase in the material toughness.

## 1. Introduction

A sheet molding compound (SMC) is a high-strength composite material comprising primarily of a thermosetting resin, filler(s), and glass fiber reinforcement. SMCs are widely used in high volume applications in automotive, aerospace, electrical, construction and appliance markets. In certain applications in aerospace, electronics or transport, the intrinsic insulating nature of the plastic composites may allow a local buildup of static electricity due to phenomena such as tribocharging or irradiation, and can ultimately lead to a dielectric breakdown, giving rise to an uncontrolled, sudden electric flow, often appearing as a visible spark. This phenomenon is known as electrostatic discharge (ESD). The immediate danger of a spark ignition in a fuel tank can easily be imagined, as the highly volatile combustible fuel vapors can ignite and give rise to explosions. One possible way to overcome this problem is to add a conductive filler such as graphene to the glass fiber–resin mixture. A considerable amount of research has been conducted on the fabrication of polymer composites using graphene and its derivatives [1,2,3,4]. Most of these studies are based on graphene produced by plasma, hummer, or chemical vapor deposition methods. These techniques are either highly energy-consuming, dangerous, or not environmentally friendly, and accordingly not suitable for mass production. Poor scalability restricts the possible applications of graphene despite its remarkable properties [5]. Mechanochemical exfoliation of graphite is a lower-cost method enabling the production of graphene nanoplatelets (GNP) on a large scale. To date, a significant amount of research has been conducted on the area of matrix modification by GNP, with remarkable outcomes. For instance, GNP-filled polymers showed improved mechanical [6], electrical [7], gas barrier [8] and heat conduction [9] properties. Moreover, recent studies on the gene toxicity of GNP have concluded that graphene is much safer than carbon black for large-scale use [10]. However, the study of the GNP/glass fiber/polymer ternary composites is limited in the literature. In particular, the effect of GNP reinforcement on sheet-mold compounding has not yet been reported. The feed material for SMCs typically consists of pre-blended polyester resin with minerals such as Calcium Carbonate (CaCO_3_) as filler.

The aim of this paper is to report progress made in the development of a new industrially-applicable thermoset nanocomposite, capable of electrostatic dissipation with mechanical properties comparable to, or better than, a conventional glass fiber reinforced composite. The matrix was a polyester resin-CaCO_3_ suspension and GNPs were used to alter the physical and mechanical properties of the matrix. It is generally accepted that a uniform dispersion of nanofillers such as GNP in polymer matrix, and ultimately in the final composite product, is essential for enhancement of properties [11]. In glass fiber-reinforced composites, even after optimized dispersion of fillers in liquid resin, nanoparticle accumulation may still occur during the manufacturing process. For instance, filler accumulation in regions close to the resin inlet compares to middle and downstream sections, results in significant anisotropy and heterogeneity [12]. In this study, we have also quantified the quality of dispersion of GNPs in the matrix by measuring the electrical conductivity of the composites in two perpendicular directions, which has also been confirmed by morphological examinations.

## 2. Materials and Methods

### 2.1. Materials

The composites are based on unsaturated polyester resin filled with ~33% CaCO_3_ (5–10 µm) (according to the technical data sheet). The role of CaCO_3_ was to reduce the material cost as well as to control shrinkage during the curing of the resin. The polyester suspension and the curing agent (an organic peroxide) were mixed with glass fibers and graphene nanoplatelets (GNP).

This new class of carbon is a middle ground between monolayered, research-grade graphene and high-thickness, multi-layered graphite, and has gained its own commercial distinction [13]. The commercially available GNPs used in this paper are GrapheneBlackTM3X from NanoXplore (Montreal, Quebec, Canada), consisting in small stacks of 6–10 graphene layers, thus falling on the nanometric scale, with an average lateral agglomerate dimension of 38 µm. 

A continuous glass chopped strand mat of randomly orientated, short-length fibers was used as the reinforcing fibers. This choice was motivated by the low cost/quality ratio of these glass fibers (GF) and their higher permeability for the resin/graphene mixture than unidirectional glass fibers.

### 2.2. Sample Preparation

Graphene-based composites with six different graphene concentrations, 0 (unfilled resin), 1, 3, 5, 7 and 10 wt.% with and without reinforcing GF were prepared. The first fabrication step was the preparation of the graphene/polyester mixture through high shear mixing at a high rotation speed. In order to keep the temperature below the decomposition temperature of the resin, the mixtures with the highest viscosity were kept in an ice bath during the mixing process. Then, the graphene/polyester mixtures were slowly poured into a mold, with or without approximately 50 wt.% of GF, and placed between the plates of a press, pre-heated at 65 °C. An initial pressure (called the kiss pressure) of 0.8 MPa was applied, followed by repeated opening-and-closing of the press to release trapped air. The pressure was then kept stable at 0.8 MPa for 30 min to allow the cross-linking initiation and completion while under pressure. Rectangular and circular specimens were cut into the molded plates for mechanical and electrical testing, respectively.

### 2.3. Morphology

The morphology and the dispersion state of GNPs were examined using scanning electron microscopy (SEM SU-8230, Hitachi, Tokyo, Japan), operating at 5 kV. The samples were coated with 3 nm of gold to improve image quality. Energy-dispersive X-ray spectroscopy (EDX, Bruker XFlash 430 H detector, Berlin, Germany) was performed at 15 kV. 

### 2.4. Electrical Characterization

Electrical properties of the composites were determined using Broadband Dielectric Spectroscopy (BDS; Novocontrol Technologies, Montabaur, Germany). The in-plane DC conductivity was measured using a home-made Four Point Probe setup with a needle spacing of 2.6 mm. A Keithley 237 instrument was used to pass a known current through the two outer probes, while an Agilent 3458A voltmeter measured output voltage across the inner probes.

### 2.5. Mechanical Tests

Three-point bending tests were conducted on rectangular samples according to the ASTM D7264 standard with the crosshead rate following the standard ASTM D790 procedure A, while impact tests were conducted on notched specimens according to the ASTM D256 standard. The dynamic mechanical properties of each composite were determined using a DMTA V (Rheometric Scientific, Piscataway, NJ, USA), tests which were performed at a frequency of 1 Hz with a temperature ramp of 3 °C/min.

## 3. Results and Discussion 

### 3.1. Electrical Properties 

**Figure 1:**
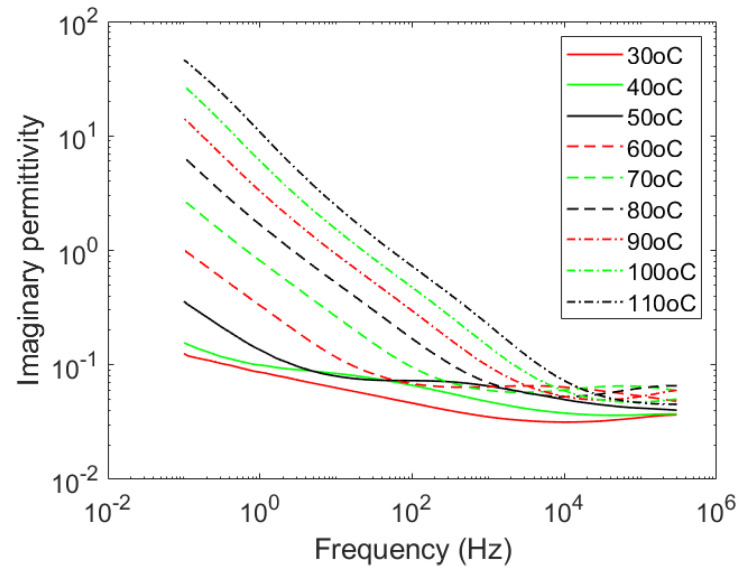
Imaginary part of the relative permittivity of unfilled fully-cured polyester resin at temperatures from 30 °C to 110 °C.

Polyester is a polar polymer with a relatively low glass transition temperature (T_g_). Figure 1 shows the isothermal curves of the imaginary permittivity of pure polyester (fully cured) at temperatures from 30 °C to 110 °C. The α relaxation peak can be readily observed in the 30 to 70 °C isothermal curves, in the form of a relaxation peak moving rapidly (high activation energy) toward high frequencies as the temperature is increased. Above glass transition, the contribution of charge fluctuations dominates the dielectric response. The low activation energy sub-glassy relaxation peak, the so-called β relaxation peak, can hardly be observed, except for a slight occurrence at the high-frequency end of the isothermal curves at 30 °C and 40 °C.

**Figure 2:**
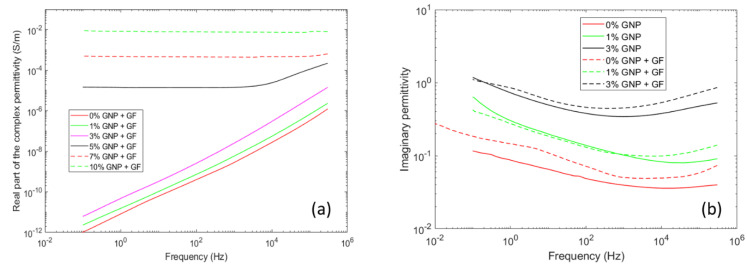
(**a**) real part of the complex conductivity of glass fiber-reinforced (GF) graphene/polyester composites; (**b**) Imaginary part of the permittivity as a function of frequency for the non-percolating composites.

The in-plane conductivity and the transverse conductivity were measured using a four-point probe system and frequency-domain spectroscopy (FDS), respectively. The FD technique enables monitoring of the complex conductivity as a function of frequency, the complex conductivity being defined by

(1) $$\hat{\sigma }\left(\omega \right)=\sigma^{\prime }\left(\omega \right)+j\sigma \prime \prime \left(\omega \right)=\omega \varepsilon_{o}\varepsilon \prime \prime \left(\omega \right)+j\omega \varepsilon_{o}\varepsilon \prime \left(\omega \right)$$

with the imaginary part of the relative permittivity (ε’’) comprising dielectric losses due to DC conductivity, and other sources of energy dissipation, such as dipolar losses, and interfacial polarization losses. Accordingly, the real part of the complex conductivity (σ’) at low frequency is equivalent to the DC conductivity, as soon as the DC conductivity contribution dominates the low frequency dielectric response, producing the typical low frequency plateau in the complex conductivity curves, as can be seen in Figure 2a. Below percolation, the inclusion of the conductive filler has the effect of increasing the dielectric loss almost uniformly in the 10^−1^ to 10^5^ Hz frequency range, as can be seen in Figure 2b. This common, although intriguing, behavior can possibly be explained by the superposition of a number of interfacial relaxation peaks that do not lead to a single relaxation peak process characterized by a distinct relaxation peak, but rather to a broad increase in the dielectric losses.

**Figure 3:**
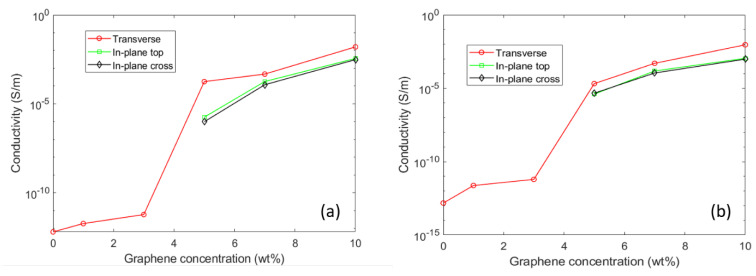
Transverse and in-plane conductivity of (**a**) unfilled and (**b**) GF-reinforced polyester as a function of the graphene concentration. The transverse conductivity was assumed to be the real part of the complex conductivity at 0.1 Hz while the in-plane conductivity was measured by the four-point technique. The latter measurement was conducted both on top of the flat section (top) and on the side section (cross) of the samples, yielding similar results.

The percolation behavior, which is characterized by a rapid increase in the direct current electrical conductivity when the conductive filler starts to form a continuous network of connected paths through the bulk of the matrix, can clearly be observed between 3 and 5 wt.%. Figure 3 shows the dependency of conductivity on graphene concentration for both types of material, with and without GF. The presence of GF was not found to significantly affect the electrical behavior of the materials. This result is somewhat remarkable, since the percolating concentration is lower than what was found for thermoplastic composites such as Ethylene-Vinyl Acetate/GNP, and not much different from the best results obtained from thermoplastic blends with a selective location of the graphene platelets [14,15]. Surprisingly, and contrary to what is usually observed in thermoplastic/graphene films [5,16], the transverse conductivity was found to be higher than the in-plane conductivity as seen in Figure 3a,b. This is certainly related to the processing technique, as well as to the relative thickness of the samples (~3 mm), and does not lead to an in-plane preferential orientation of the graphene nanoplatelets. As described earlier, fiber textiles can act as macro-sized mesh membranes, resulting in the filtration of nanoparticles with larger surface areas. The isotropic electrical behavior of GF-reinforced samples suggests that the glass fiber almost had no filtering effect on these suspensions.

### 3.2. Mechanical Properties

**Figure 4:**
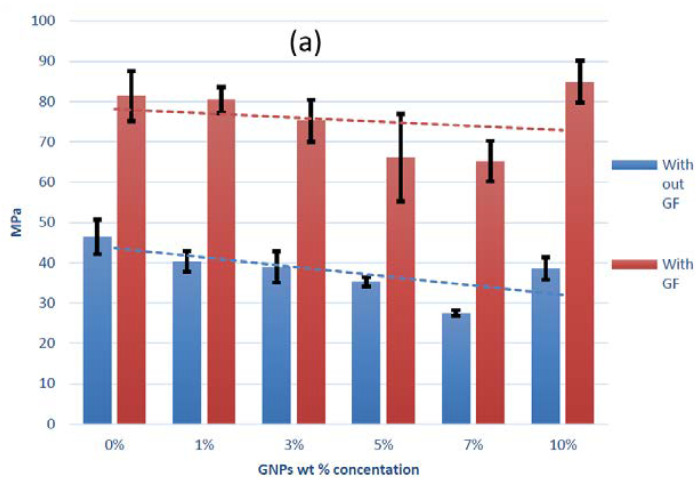

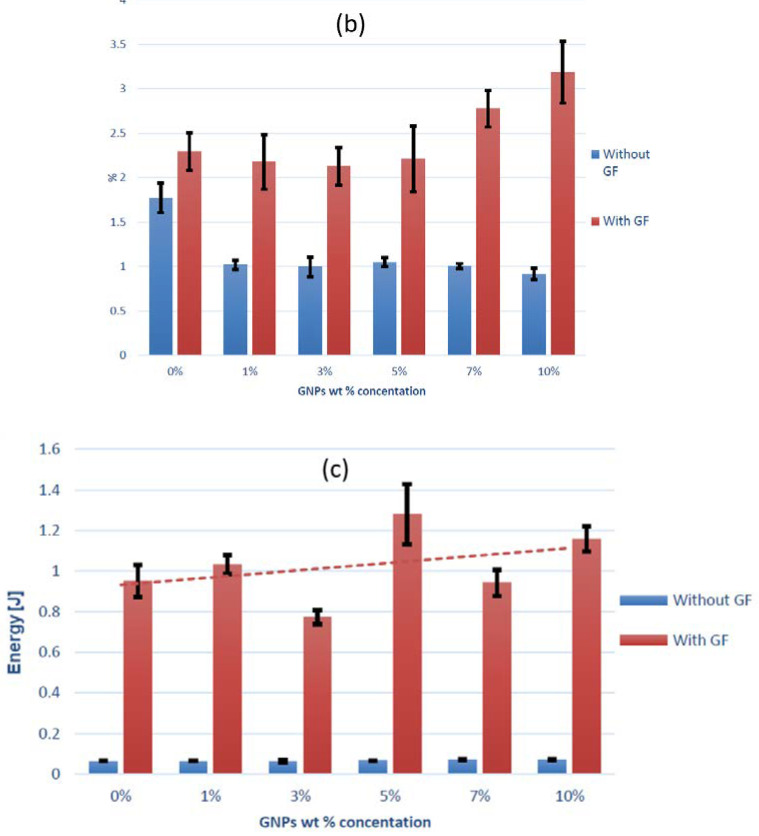
Mechanical properties of unfilled and GF-reinforced polyester as a function of graphene content. (**a**) Flexural stress-at-break; (**b**) flexural strain-at-break; (**c**) absorbed impact energy.

Three-point bending and impact tests were conducted on all samples, with and without glass fibers. The results of both tests are shown in Figure 4. As can be seen, the inclusion of graphene was not found to have a significant impact on the mechanical properties of either unfilled or GF-reinforced polyester, except maybe a slight increase in toughness and strain-at-break for the GF-reinforced composites. 

**Figure 5:**
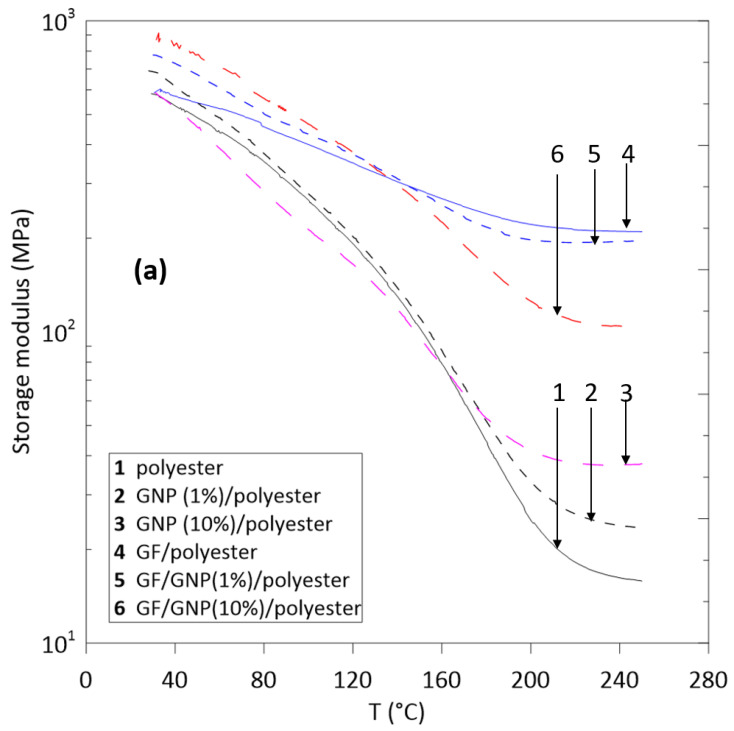

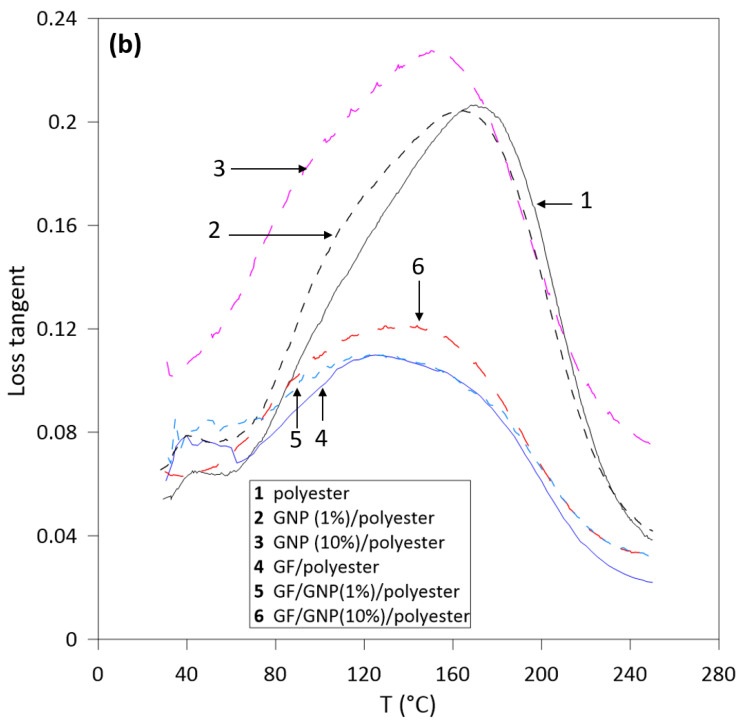
(**a**) Storage modulus and (**b**) loss tangent values of the glass fiber/polyester and GNPs (graphene nanoplatelets)/glass fiber/polyester composites.

Figure 5 presents dynamic mechanical analysis (DMA) measurements on GF/polyester and GNP/GF/polyester composites, which shows the temperature dependence of storage modulus (*E’*) and loss tangent (tan δ), at a single test frequency *f* = 1 Hz. As the temperature increased, the *E’* decreased sharply until reaching the robbery plateau (Figure 5a); the difference between glass and rubber moduli is much smaller in the GF- reinforced composites. At lower temperatures (T < 150 °C), the maximum improvement in *E’* was found in the case of GNP/GF/polyester composites, which was similar to the trend observed for flexural modulus. It could be due to stronger interfacial interactions: between GNP and glass fiber; GNP and the matrix; fiber and the matrix. At higher temperatures, however, the highest dynamic modulus was found in the case of GF/polyester composite without GNP.

Figure 5b shows that the temperature corresponding to the tan δ maximum peak did not change significantly with GNP loading. Due to the relatively broad α-relaxation temperature range of these composites in mechanical loss spectra, it would be inappropriate to choose the temperature corresponding to the tan δ peak as the T_g_ value. As well as the α-relaxation peak around T_g_, one can see that the β relaxation peak occurs in the temperature range of 30 °C–50 °C. This peak could be associated with the high-frequency β relaxation in dielectric spectra, although it should be considered that there are significant differences between the relaxation behavior determined from dielectric and dynamic mechanical data [17]. 

As shown in Figure 5b, for the composites containing high amounts of GNP, β relaxation appears to be absent; instead, an excess contribution to the tail of the α relaxation is observed.

**Figure 6:**
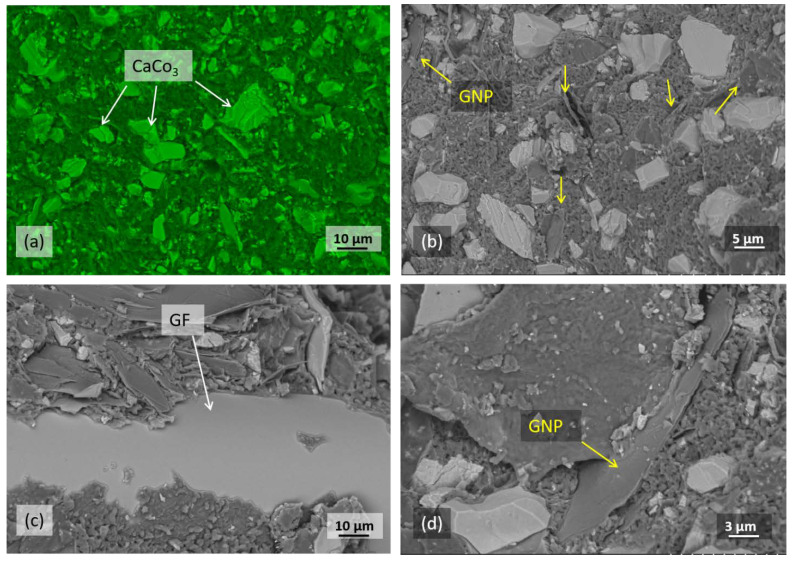
SEM (scanning electron microscopy) images of the composites; (**a**) blend of polyester and CaCO_3_, (**b**) the same blend mixed with 5 wt.% GNP, (**c**) GF-reinforced mixture and (**d**) interfacial adhesion between resin and GNP. Backscattering electron signals were employed to help us to differentiate between CaCO_3_ and GNP.

**Figure 7:**
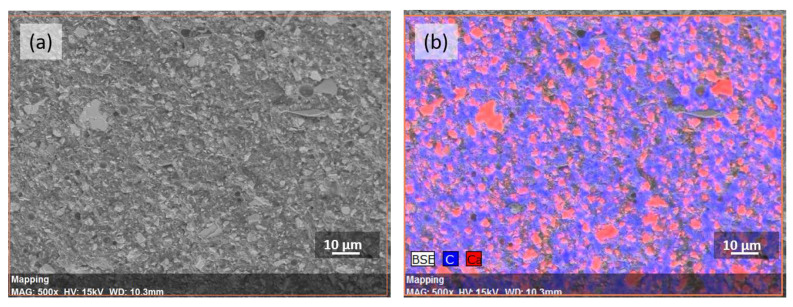
SEM image (**a**) and the corresponding EDX (Energy-dispersive X-ray spectroscopy) mapping image (**b**) of the elements C and Ca. The colours blue and red indicate the distribution of carbon and calcium in the sample, respectively.

To study the interfacial adhesion between the components and matrix material, SEM was performed. The fracture surface of the non-reinforced polyester system displays a relatively smooth surface indicating a brittle-type failure (Figure 6a,b). It can be seen that CaCO_3_ and GNP are both properly dispersed in the resin. The atomic composition of the polyester suspension is confirmed by EDX (Figure 7). A complete uniformity is required, otherwise areas of non-interaction can occur, decreasing the strength of the composite. To investigate the adhesion between GF, GNP and the matrix, the morphologies of the GNP/GF/polyester composites are shown in Figure 6c,d. According to Figure 6c, traces of the matrix were found stuck to the surface of fibers, which clearly reveals a good interfacial adhesion between the glass fiber and matrix material. Figure 6d also presents a relatively good affinity between GNP and the matrix phase.

## 4. Conclusions

Electrically conductive nanocomposites and glass fibre-reinforced composites were produced based on polyester resin blended for SMC applications. Industrially-produced GNP was added to the resin as the conductive filler. The percolation threshold was observed between 3 and 5 wt.%, with the conductivity increasing by more than 7 orders of magnitude in both cases. Similar in-plane and cross-plane electrical conductivity values indicated a reasonable dispersion of GNPs in these composites. Except for a slight increase in the material toughness, the composites showed no significant change of mechanical properties due to the introduction of GNPs when compared to the non-filled polyester/glass fiber composite. At lower temperatures, the ternary GNP/GF/polyester composites demonstrated a higher storage modulus than that of the binary GF/polyester composites, which could be attributed to good GNP/GF or GNP/matrix interfacial interactions. The low amount of filler needed, in conjunction to the scalability potential and simplicity of the process, would enable the large-scale producibility of this type of composite.
